# Patterns of HER2 Gene Amplification and Response to Anti-HER2 Therapies

**DOI:** 10.1371/journal.pone.0129876

**Published:** 2015-06-15

**Authors:** Rocio Vicario, Vicente Peg, Beatriz Morancho, Mariano Zacarias-Fluck, Junjie Zhang, Águeda Martínez-Barriocanal, Alexandra Navarro Jiménez, Claudia Aura, Octavio Burgues, Ana Lluch, Javier Cortés, Paolo Nuciforo, Isabel T. Rubio, Elisabetta Marangoni, James Deeds, Markus Boehm, Robert Schlegel, Josep Tabernero, Rebecca Mosher, Joaquín Arribas

**Affiliations:** 1 Preclinical Oncology Program, Vall d’Hebron Institute of Oncology (VHIO), Universitat Autònoma de Barcelona, 08035, Barcelona, Spain; 2 Clinical Oncology Program, Vall d’Hebron Institute of Oncology (VHIO), Universitat Autònoma de Barcelona, 08035, Barcelona, Spain; 3 Molecular Oncology Program, Vall d’Hebron Institute of Oncology (VHIO), Universitat Autònoma de Barcelona, 08035, Barcelona, Spain; 4 Pathology Department, Vall d’Hebron University Hospital, 08035, Barcelona, Spain; 5 Pathology Department, Hospital Clínico Universitario, INCLIVA Biomedical Research Institute, 46010, Valencia, Spain; 6 Oncology Department, Hospital Clínico Universitario, INCLIVA Biomedical Research Institute, 46010, Valencia, Spain; 7 Translational Research Department, Institut Curie, 75005, Paris, France; 8 Novartis Oncology Translational Research, Cambridge, MA, 02139, United States of America; 9 Novartis Pharma AG, Postfach, CH-4002, Basel, Switzerland; 10 Department of Biochemistry and Molecular Biology, Universitat Autònoma de Barcelona, Campus de la UAB, 08193, Bellaterra, Spain; 11 Institució Catalana de Recerca i Estudis Avançats (ICREA), 08010, Barcelona, Spain; University of Torino, ITALY

## Abstract

A chromosomal region that includes the gene encoding HER2, a receptor tyrosine kinase (RTK), is amplified in 20% of breast cancers. Although these tumors tend to respond to drugs directed against HER2, they frequently become resistant and resume their malignant progression. Gene amplification in double minutes (DMs), which are extrachromosomal entities whose number can be dynamically regulated, has been suggested to facilitate the acquisition of resistance to therapies targeting RTKs. Here we show that ~30% of HER2-positive tumors show amplification in DMs. However, these tumors respond to trastuzumab in a similar fashion than those with amplification of the HER2 gene within chromosomes. Furthermore, in different models of resistance to anti-HER2 therapies, the number of DMs containing HER2 is maintained, even when the acquisition of resistance is concomitant with loss of HER2 protein expression. Thus, both clinical and preclinical data show that, despite expectations, loss of HER2 protein expression due to loss of DMs containing HER2 is not a likely mechanism of resistance to anti-HER2 therapies.

## Introduction

Increase in the number of copies of a chromosomal region is known as amplification and it is a frequent mechanism by which proto-oncogenes are transformed into oncogenes. Approximately 15–20% of breast cancers are characterized by the amplification of a region located in chromosome 17. This region (17q12q21) contains the proto-oncogene HER2, a receptor tyrosine kinase that belongs to the family of the epidermal growth factor receptor (EGFR), whose overexpression is considered a potent tumor driver [1,2].

Gene amplification is likely initiated by DNA double-strand breaks and it occurs only in cells endowed with the ability to progress through the cell cycle carrying damaged DNA. Although the mechanism(s) that lead to gene amplification remain(s) largely unknown, the final distribution of amplified DNA has been characterized in some detail. Amplified DNA can form tandem arrays, as head-to-tail or head-to-head repeats, within a chromosome. These repetitions are cytologically visible as homogeneously staining regions (HSRs). Alternatively, amplified DNA can be stored in extrachromosomal entities called double minutes (DMs). While HSRs follow the same fate as the rest of chromosomal regions during mitosis, DMs do not contain centromeres, do not bind the mitotic spindle and, thus, are likely not distributed evenly between daughter cells [3].

Currently, HER2-amplified breast cancers are treated with monoclonal antibodies against the tyrosine kinase receptor such as trastuzumab, alone or coupled to emtansine, a cytotoxic agent (T-DM1). An alternative therapy is based on synthetic tyrosine kinase inhibitors, such as lapatinib. Despite the remarkable effectiveness of these therapies, tumors frequently become resistant to them and resume their malignant progression [4].

The type of gene amplification may influence the effectiveness of targeted therapies. Many glioblastomas, the most common type of brain tumor, are characterized by the amplification in DMs of EGFR-vIII, a gene encoding a constitutively active form of EGFR [5]. Upon treatment with tyrosine kinase inhibitors, glioblastoma cells survive by losing DMs and, thus, downmodulating the expression of EGFR-vIII [6]. Upon removal of the drug, resistant cells regain EGFR-vIII gene copies by re-accumulating DMs. Similar dynamic control of protein expression, through the elimination or accumulation of DMs, has been shown in different models including cells with amplified Myc [7] or dihydrofolate reductase [8].

Preliminary results have shown that resistance to anti-HER2 therapy can be caused by loss of HER2 expression [9,10]. However, since the patterns of amplification of HER2 have not yet been characterized, it is not known whether loss of DMs may be involved in the acquisition of resistance to anti-HER2 therapies.

Here, we show that HER2 is amplified in DM or in HSR regions in ~30 and ~60% of HER2-positive breast tumors, respectively. Despite expectations, the response of breast tumors with HER2 amplified in DM to anti-HER2 therapies is similar to that of tumors with HSR. Furthermore, using preclinical models of resistance to different anti-HER2 therapies (trastuzumab, trastuzumab-emtansine (T-DM1) and lapatinib), we show that, even when acquisition of resistance is concomitant with loss of HER2 protein expression, it occurs without loss of DMs.

## Materials and Methods

### Cell Lines

HCC1954, MDA-MB-453, SkBr3 and BT474 cells were from ATCC-LGC Standards (Teddington, UK). SkBr3 and BT474 were maintained in Dulbecco's Modified Eagle's Media (DMEM):F-12, HCC1954 in RPMI 1640 and MDA-MB-453 in Leibovitz’s L-15, all 10% FBS and 4 mmol/L L-glutamine (all from Gibco).

### Animal Research

The study was performed in accordance with European Community Standards of Care and Use of Laboratory Animals. Approval was granted for the animal experiments by the Vall d'Hebron University Hospital Care and Use Committee.

### Tumor Samples

Human breast tumors used in this study were from biopsies or surgical resections at Vall d'Hebron University Hospital, Barcelona and Hospital Clinico Universitario, Valencia (Spain) and were obtained following institutional guidelines. The institutional review boards (IRB) at Vall d’Hebron Hospital and Hospital Clinico de Valencia provided approval for this study in accordance with the Declaration of Helsinki. Written informed consent for the performance of tumor molecular studies was obtained from all patients who provided tissue. The baseline characteristics of the two cohorts of patients used in this study (neo-adjuvance and adjuvance cohorts) are shown in S1 and S2 Tables.

### DISH / Determination of HER2 Amplification Patterns

The INFORM HER2 Dual ISH DNA Probe Assay was used on 5-μm sections using the BenchMark XT Staining Platform (Ventana Medical Systems). All samples were processed following the FDA-approved protocol. Samples with >70% of the cells with a DM (small dispersed dots distributed throughout the nucleus) or HSR (tightly clustered dots in discrete regions of the nucleus) patterns were classified accordingly. Cases not falling into these categories because of the presence of both HSR and DM patterns in the same sample were classified as mixed; for example, samples with 40% and 60% of cells with DM and HSR patterns, respectively, were classified as mixed while samples with 20% and 80% of cells with DM and HSR patterns, respectively, were classified as HSR (see text for details). Two teams including certified pathologists led by V. P. and R. M. blindly assessed the pattern of HER2 amplification in the series of samples from patients treated with neoadjuvant trastuzumab. The pattern of amplification in the series of samples from patients treated with adjuvant trastuzumab was assessed by V. P. The HER2/centromere 17 probe signal ratio was determined based on the quantification of 20 cells in at least two different fields, as recommended in ASCO/CAP 2013 guideline [11].

### DISH or Metaphase Spreads on Cells Treated with Colchicine

Cells grown to 85% confluence were treated with 100 ng/ml of colchicine (Gibco) for 18 hours, trypsinized and pelleted. Then, cell pellets were stained by DISH or treated with an hypotonic solution (0.075 M KCl) at 37°C for 15 minutes. After the hypotonic treatment, cells were treated with ice-cold fixative (1:3 acetic acid:methanol). Metaphase spreads were obtained by dropping fixed cells onto slides. Slides were air-dried overnight and then stained with Giemsa.

### Patient-Derived Xenografts (PDX)

Six to eight-week old NOD.CB17-Prkdc^scid^/J (NOD/SCID) mice were purchased from Charles River Laboratories. Mice were maintained and treated in accordance with institutional guidelines of Vall d’Hebron University Hospital Care and Use Committee. Fragments of patient samples were implanted into the fourth fat pad of the mice, which were supplemented with 17 ß-estradiol (1 μM) (Sigma-Aldrich) in the drinking water [12]. PDX models 288, 289 and 290 corresponded to HBCx-13B, HBCx-41and HBCx-5, respectively, and were established at Institut Curie as previously described [13]. Written informed consent was obtained from all patients who provided tissue.

### Establishment of Cell Cultures from PDXs

For the establishment of cell cultures derived from PDXs, tumors were excised and cut into the smallest pieces possible with scalpel, incubated for 30 minutes with collagenase IA (Sigma-Aldrich), washed and resuspended in DMEM:F-12, 10% FBS, 4 mmol/L L-glutamine, Penicillin/Streptomycin (Gibco), 10 mM HEPES (Santa Cruz Biotechnology) and 1.75 μg/ml Amphotericin B (Gibco) for 6 hours. Then, medium was carefully removed and changed to 10%-supplemented Mammocult human medium (StemCell Technologies) with Penicillin/Streptomycin, 10 mM HEPES and 1.75 μg/ml Amphotericin B for one week in order to facilitate the growth of epithelial cells with respect to contaminating mouse fibroblasts.

### Immunohistochemisty (IHC)

IHC was performed on FFPE PDX samples with HerceptTest (Dako) according to the manufacturer’s instructions. A certified pathologist, C.A, evaluated HER2 protein expression by H-score.

### qPCR

RNA was isolated from FFPE tumors or cell pellets with QIAamp DNA FFPE Tissue Kit (QIAGEN) following manufacturer’s instructions. Ten ng of DNA were run in a Applied Biosystems 7900HT using ErbB2 TaqMan Copy Number Assays (Hs00641606_cn) and RNase P TaqMan Copy Number Reference Assay (4403326). Each sample was assayed in quadruplicate. Copy number was calculated with Applied Biosystems CopyCaller Software following manufacturer’s instructions.

### Cell Proliferation

Cells were plated and treated with lapatinib or T-DM1 at the described concentrations. At the indicated time points, cells were fixed by replacing the growth medium with 100 μl/well of 10% glutaraldehyde in phosphate-buffered saline (PBS) for 10 minutes at RT. After three washes with water, 100 μl of 0.1% crystal violet solution was added to each well and incubated 20 minutes. Plates were washed and air dried, and 50 μl of 10% acetic acid was added to each of the wells. Optical Density (OD) was measured at 560 nm.

### Western Blot

Western blot was carried out as previously described [14]. The following antibodies were used: mouse monoclonal anti-HER2 (BioGenex, CB11), rabbit polyclonal anti-β-Tubulin (Santa Cruz, H-235) and rabbit polyclonal anti-GAPDH (Trevigen). Densitometry quantification was performed using ImageJ software.

### Statistical Analysis

The *P* values were calculated by the two-sided Fisher’s exact test and the Mantel-Cox test using the GraphPad Prism version 5.0 software.

## Results

### HER2 Amplification Patterns in Breast Cancer Cells

**Fig 1:**
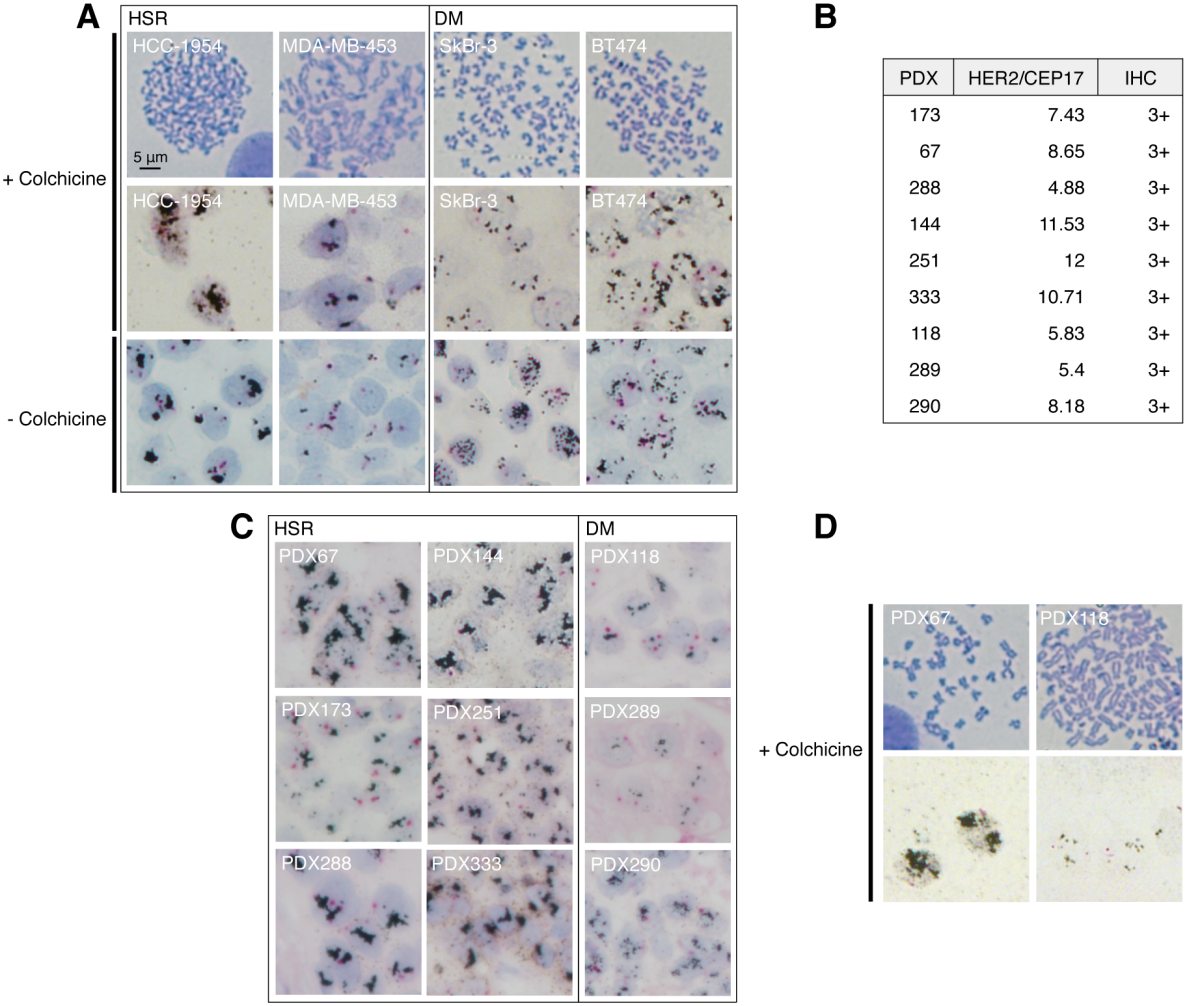
HER2 gene amplification patterns in different breast cancer cell lines and PDXs. A, The indicated cell lines were treated with colchicine and metaphase spreads (top panels) or HER2 DISH staining (middle panels) were performed as described under Materials and Methods. As a control, HER2 DISH was also performed on untreated cultures of the same cell lines (lower panels). B, Samples from the indicated PDXs were analyzed by HER2 DISH and the pattern of gene amplification as well as the ratio HER2/CEP17 were determined. Levels of HER2 protein expression were analyzed by immunohistochemistry (IHC). C, Samples from the indicated PDXs were analyzed by HER2 DISH. D, Cell cultures obtained from PDXs with HSR or DM HER2 gene amplifications were treated with colchicine and metaphase spreads (upper panels) or HER2 DISH (lower panels) were performed as in A.

Analysis by dual-ISH (DISH) of HER2-amplified breast cancer cell lines treated with colchicine, which arrests cells in metaphase, showed two patterns: tightly clustered dots in discrete regions or many small dispersed dots distributed throughout the nucleus (Fig 1A, +Colchicine). The first pattern, also known as homogeneously staining region (HSR), corresponds to tandem gene amplification, the second pattern is consistent with gene amplification in double minutes (DM). Analysis of interphase cells showed a pattern similar to that observed in metaphase cells (Fig 1A, -Colchicine), indicating that gene amplification patterns can be identified in conventionally prepared tumor samples.

We confirmed the existence of HER2 gene amplifications in HSR and DM in nine patient-derived xenografts (PDX) (Fig 1B and 1C). To validate these patterns, we cultured cells from PDXs with HSR or DM amplifications, and performed DISH on cells treated with colchicine. As shown in Fig 1D, we observed similar patterns, further supporting that the type of HER2 gene amplification pattern is identifiable in clinically relevant samples.

We concluded that, in breast cancers, HER2 can be amplified both in HSR or DM patterns.

### HER2 Amplification Patterns and Response to Trastuzumab

**Fig 2:**
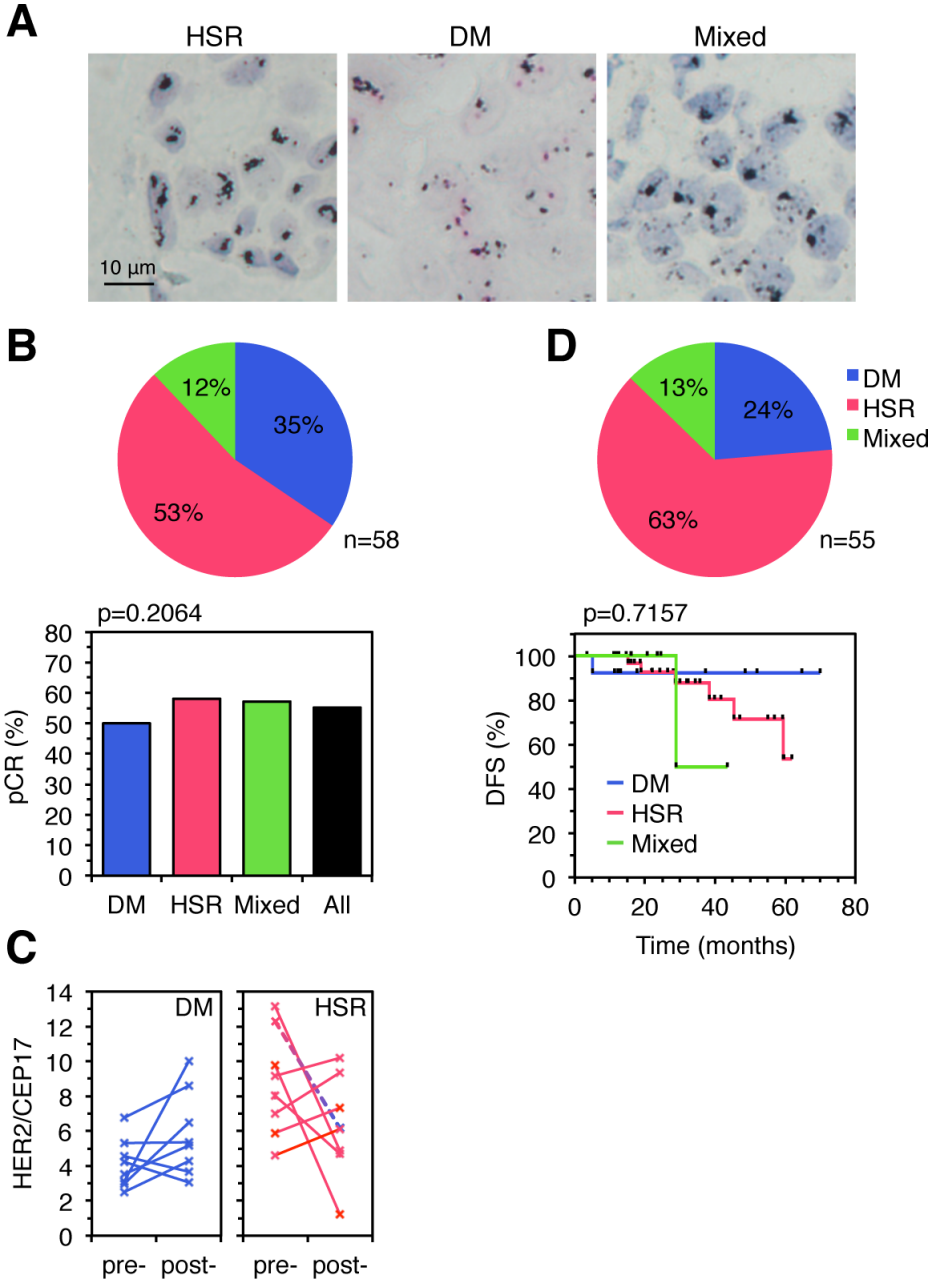
HER2 amplification patterns and response to trastuzumab. A, The pattern of amplification of samples from two cohorts of breast cancers patients treated with neoadjuvant or adjuvant trastuzumab was determined as in Fig 1A and samples were classified as HSR, DM or mixed (see text for details). Representative examples are shown. B, Upper graph, percentages of the HER2 gene amplification patterns in tumors from a cohort of breast cancer patients treated with neoadjuvant trastuzumab. Lower graph, pathologic complete response (pCR) rates according to the HER2 gene amplification pattern. *P* values shown were calculated by the two-sided Fisher’s exact test. C, Paired samples, pre- and post-treatment with trastuzumab, from tumors with DM and HSR amplifications, obtained from patients without pCR, were analyzed by HER2 DISH and the ratio HER2/CEP17 was calculated and represented. D, Upper graph, percentages of the HER2 gene amplification patterns in tumors from a cohort of breast cancer patients treated with adjuvant trastuzumab. Lower graph, disease-free survival (DFS) according to HER2 gene amplification pattern. *P* values shown were calculated by the Mantel-Cox test comparing the HSR and DM groups.

The analysis of 58 samples from HER2-amplified tumors treated with trastuzumab plus chemotherapy prior to surgical resection (neoadjuvant treatment) showed that 35% and 53% had DM and HSR patterns, respectively. The rest of samples contained both patterns and were considered mixed (Fig 2A and 2B, upper graph). Consistent with previous reports [15], treatment led to pathological complete response (pCR) in approximately 55% of the cases (Fig 2B, lower graph). The percentage of pCR in the DM group (50%) was not statistically different from that of the HSR group (57%).

Samples from 16 cases (8 with DM and 8 with HSR amplification) without pCR were analyzed pre- and post treatment. The average amplification ratios of the cases with DM, pre- and post-treatment, were 4.10 and 5.82, respectively (S3 Table and Fig 2C), showing that the cases with DM that did not achieve pCR did not tend to lose HER2 gene copies. In contrast, the average amplification ratios of the pre- and post-treatment cases with HSR were 8.72 and 6.25, respectively. The pattern of amplification did not change with treatments in all cases but one, in which we observed a transition from HSR to DM (dotted line in Fig 2C). Thus, these results do not support that treatment with trastuzumab results in loss of HER2 gene copies from tumors with DM amplification or in a change in the amplification pattern.

To extend these results, we analyzed the pattern of HER2 amplification in tumors from a cohort of patients (n = 55) treated after surgery with trastuzumab (adjuvant treatment). In agreement with the results in the neoadjuvant setting, the percentage of tumors with DM amplification that progressed was not significantly different from that of tumors with HSR amplification (Fig 2D).

Thus, the amplification of HER2 in DM does not correlate with resistance to trastuzumab.

### Acquisition of Resistance to Therapeutic Anti-HER2 Antibodies in Tumors with DM Amplification In Vivo

**Fig 3:**
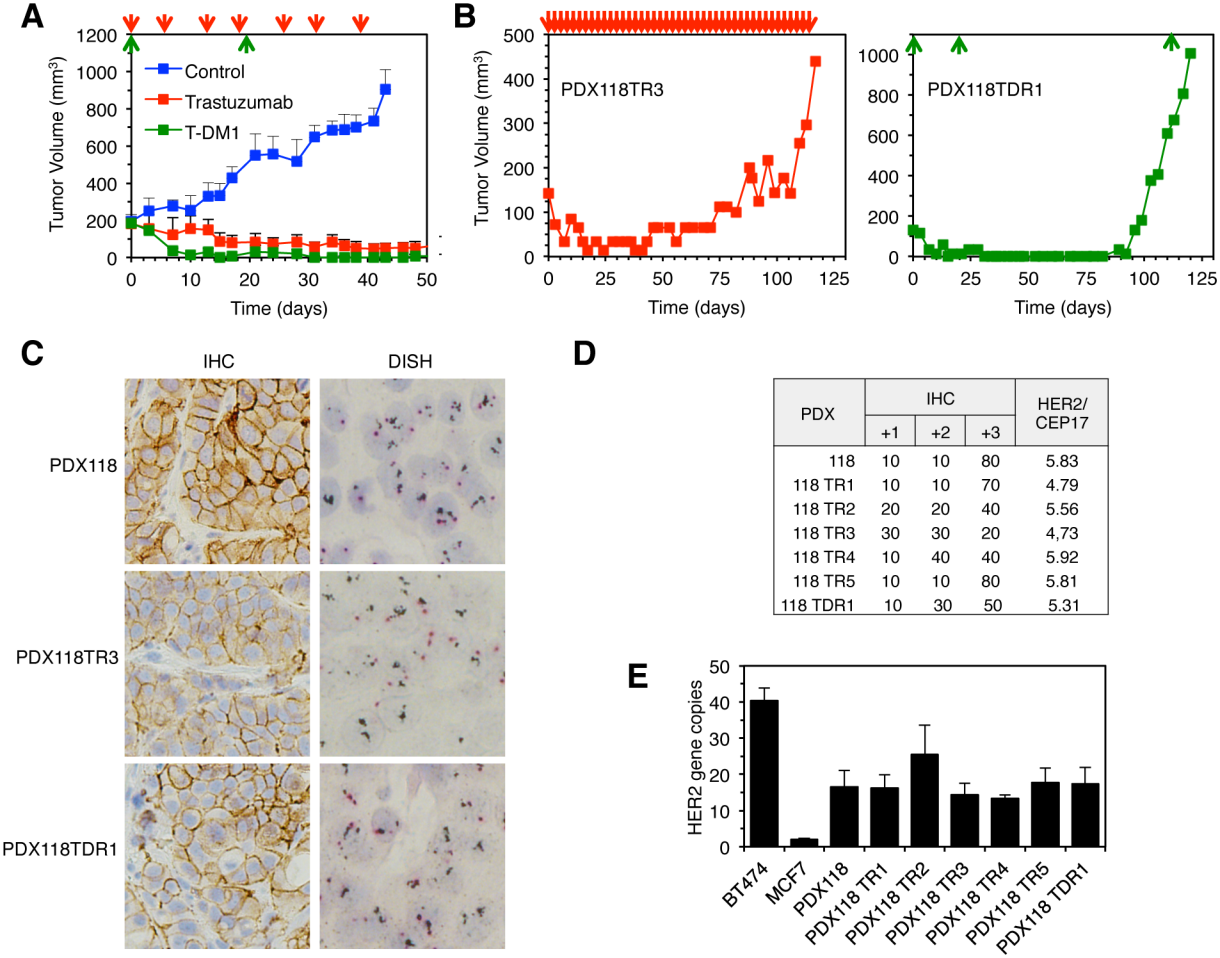
HER2 amplification pattern and gene copy number in tumors resistant to trastuzumab or T-DM1. A, NOD/SCID mice (n = 8 per group) carrying PDX118, which has amplification in DM (see S1B and S1C Fig), were treated with anti-HER2 antibodies as indicated (red and green arrowheads indicate the administrations of trastuzumab (10 mg/kg twice per week, i.p) and T-DM1 (15 mg/kg every three weeks, i.v., respectively). Tumor volumes were calculated using the formula: (length × width2) × (pi/6) and results are expressed as averages. Error bars correspond to 95% confidence intervals. B, Mice carrying PDX118 were chronically treated with trastuzumab (left) or twice with T-DM1(right) and tumor volumes were determined as in A. The growth of representative individual tumors is shown. C, HER2 protein levels and HER2 gene amplification were analyzed by immunohistochemistry and DISH, respectively, in samples from parental PDX118, or samples from the tumors shown in B. D, HER2 protein levels determined by IHC or HER2 gene amplification determined by DISH were quantified in parental PDX118 or in samples from resistant xenografts shown in B and in S2 Fig E, Gene copy number was determined by qPCR as described under Experimental Procedures.

To characterize the acquisition of resistance to anti-HER2 therapies in tumors with DM amplification, we used a PDX (PDX118, Fig 1) that is sensitive to trastuzumab and to T-DM1 (Fig 3A). After 75–125 days of continuous treatment with trastuzumab (10 mg/kg, biweekly), the tumors in five out of six mice relapsed (Fig 3B, TR stands for trastuzumab resistant and S1 Fig) and their resistance to trastuzumab was confirmed after re-implantation into new mice (S2 Fig).

T-DM1 effectively reduced the size of tumors to undetectable levels after two dosages (Fig 3A). ~70 days after treatment, tumors regrew in four out of six mice (Fig 3B, TRD stands for T-DM1 resistant and data not shown) and one of these tumors was confirmed as resistant to T-DM1(S2 Fig).

All the resistant tumors but one (PDX118 TR5) showed decreased expression of HER2 (Fig 3C and 3D and S3 Fig). Thus, acquisition of resistance to anti-HER2 therapies in this model of DM amplification can be concomitant with loss of HER2 protein expression.

The HER2 / CEP17 ratios of these samples indicated a similar level of amplification of HER2 in the parental and resistant tumors (Fig 3D). We confirmed these data by a qPCR-based method (Fig 3E) and concluded that the downregulation of HER2 protein in the trastuzumab and T-DM1 resistant tumors is not due to a decrease in HER2 gene copy number.

### Acquisition of Resistance to Anti-HER2 Therapies In Vitro

**Fig 4:**
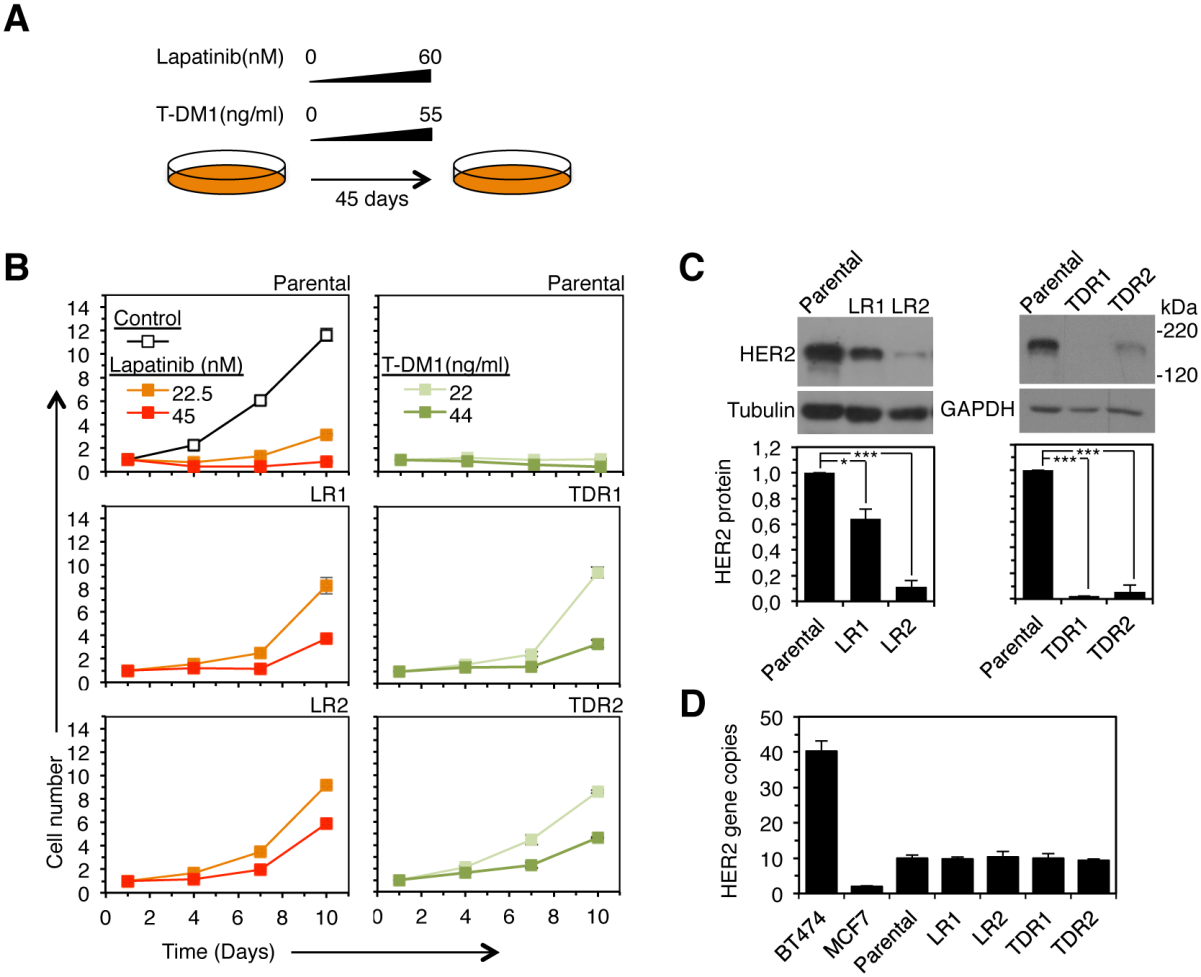
HER2 amplification pattern and gene copy number in cells resistant to lapatinib or T-DM1. A, Schematic showing the procedure to obtain cells resistant to anti-HER2 therapies *in vitro*. Cultures from PDX118 were treated with increasing concentrations of lapatinib or T-DM1 for 45 days. B, Parental PDX118 cell cultures or cells selected in the presence of lapatinib or T-DM1 as shown in A, in two independent experiments, were plated in the presence of the indicated concentrations of the anti-HER2 drugs. At the indicated time points, cell proliferation was determined and normalized. The results were expressed as averages ± standard deviations. C, The same cells as in B were lysed and the cell lysates analyzed by Western blot with antibodies against HER2 in three independent experiments. Representative results are shown. Blots were quantified and the results were expressed as averages ± standard deviations. *, *P* < 0.05; ***, *P* < 0.001. D, Gene copy number was determined by qPCR as described under Experimental Procedures.

The elimination of DMs carrying EGFR-vIII from glioblastoma cells can be caused by specific tyrosine kinase inhibitors [6]. Lapatinib impaired the growth of cell cultures established from PDX118 (Fig 4B, Parental). Prolonged treatment of these cell cultures with increasing concentrations of the tyrosine kinase inhibitor led to the appearance of resistant cells (Fig 4A and 4B, LR stands for lapatinib resistant). Analysis of two batches of resistant cells, generated independently, showed downregulation of HER2 (Fig 4C). However, HER2 / CEP17 gene ratios of lapatinib-resistant and parental cells were comparable (data not shown) and quantification by qPCR confirmed the maintenance of HER2 gene copy number (Fig 4D).

Similar results were obtained with T-DM1. Prolonged treatment *in vitro* with increasing concentrations of the antibody led to the appearance of resistant cells that showed a marked downregulation of HER2. However, this HER2 protein downmodulation occurred without loss of HER2 gene copies (Fig 4).

We concluded that acquired resistance of breast cancer cells to anti-HER2 therapies, even when concomitant with the loss of HER2 protein expression, is not due to the elimination of DMs encoding the tyrosine kinase receptor.

## Discussion

Overexpressed HER2 is a prototypic biomarker as well as the target of effective anti-tumor therapies. As a biomarker, overexpressed HER2 defines a subtype of breast cancers with specific biological features. As a target, therapies against HER2 have prolonged the life of breast cancer patients carrying HER2 gene amplification. Unfortunately, some HER2-positive tumors are intrinsically resistant to anti-HER2 therapies and other tumors, after an initial response, become resistant [4].

Despite the available wealth of knowledge on HER2, basic aspects of its biology are still uncharacterized. Here, we present the first study on the patterns of amplification of HER2 in breast cancers. The presence of two distinct amplification patterns (HSR and DM) (Figs 1 and 2) is indicative of different mechanisms of amplification. Although in most breast tumors the majority of the cells exhibit mostly one of the amplification types, the existence of tumors with mixed pattern of amplification indicates that different mechanisms of HER2 gene amplification may co-exist within the same tumor.

Evidence indicating that the amplification of genes in DM may result in a dynamic regulation of gene expression, through the reversible gain or lose of gene copies, has slowly but steadily accumulated [8] [7] [6]. Since resistance to anti-HER2 therapies may be caused by downmodulation of HER2 expression ([10], see also Figs 3 and 4), it was reasonable to hypothesize that amplification of HER2 in DM would endow breast cancers with the ability to modulate HER2 protein expression and, thus, sensitivity to anti-HER2 therapies by dynamically regulating HER2 copy number. Our results clearly disprove this hypothesis. On one hand, we showed that breast cancer tumors with HER2 amplification in DM respond in a similar fashion to neoadjuvant or adjuvant trastuzumab (Fig 2). Furthermore, tumors with amplification in HSR show a tendency to lose copies of the HER2 gene that is not observed in tumors with amplification in DM (Fig 2C). On the other hand, we showed that resistance against different anti-HER2 therapies (trastuzumab, T-DM1 and lapatinib) can be acquired without modifying DM content, even if it is concomitant with the loss of HER2 protein expression. In line with our conclusions, breast cancer patients with cluster amplification of HER2, treated with trastuzumab-based therapies, had shorter survival than those with non-cluster amplification [16]. Since cluster amplification likely corresponds to amplification in HSR, this result does not support that tumors with DM amplification are more likely to develop resistance against trastuzumab.

Our results suggest that, in addition to HER2, the amplicon carried by breast cancer cells includes other critical loci. The strongest indication is the maintenance of the amplified DNA even in resistant cells that have lost HER2 protein expression. Furthermore, treatment with DNA-damaging drugs, such as hydroxyurea, previously shown to induce the loss of DMs in different cells [7,17] do not have an effect on the levels of DMs in HER2-amplified breast cancer cells (data not shown). These results point to the presence of additional sequences in the amplicon of HER2 that contribute to the growth of breast cancer cells. In agreement with this conclusion, it has been shown that the co-silencing of HER2 with other genes included in the HER2 amplicon, such as STARD3, GRB7, PSMD3 and PERLD1, leads to an additive impairment of cell viability [18]. The analysis of the expression of these genes in our resistant tumors may shed light on the maintenance of cell viability in the presence of diminished levels of HER2.

## Supporting Information

S1 Fig Selection of PDXs resistant to trastuzumab. Mice carrying PDX118 were chronically treated with trastuzumab and tumor volumes were calculated as in Fig 3A. The growth of individual tumors is shown.
(TIF)

S2 Fig Validation of PDXs resistant to trastuzumab or T-DM1. Small pieces of the tumors that progressed after trastuzumab or T-DM1 treatment (see Fig 3B and S1 Fig) were orthotopically implanted into new NOD/SCID mice (n = 3 per group). Tumor volumes were determined at the indicated time points.
(TIF)

S3 Fig Analysis of HER2 protein levels and gene amplification in PDXs resistant to trastuzumab or T-DM1. Samples from the indicated tumor grafts were analyzed by immunohistochemistry with antibodies against HER2 to determine HER2 protein levels, or by DISH to determine HER2 gene amplification.
(TIF)

S1 Table Baseline characteristics of the cohort treated with neoadjuvant trastuzumab. (DOCX)

S2 Table Baseline characteristics of the cohort treated with adjuvant trastuzumab. (DOCX)

S3 Table Analysis of the HER2/CEP17 ratio in samples with amplification double minutes (DM) or homogeneously staining regions (HSR) pre- and post-treatment. (DOCX)

## References

[1] Yarden Y, Pines G (2012) The ERBB network: at last, cancer therapy meets systems biology. Nature Publishing Group 12: 553–563. 10.1038/nrc3309 22785351

[2] Arteaga CL, Engelman JA (2014) ERBB Receptors: From Oncogene Discovery to Basic Science to Mechanism-Based Cancer Therapeutics. Cancer Cell 25: 282–303. 10.1016/j.ccr.2014.02.025 24651011 PMC4018830

[3] Albertson DG (2006) Gene amplification in cancer. Trends in Genetics 22: 447–455. 10.1016/j.tig.2006.06.007 16787682

[4] Gradishar WJ (2012) HER2 therapy—an abundance of riches. N Engl J Med 366: 176–178. 10.1056/NEJMe1113641 22149874

[5] Bonavia R, Inda M-D-M, Cavenee WK, Furnari FB (2011) Heterogeneity maintenance in glioblastoma: a social network. Cancer Research 71: 4055–4060. 10.1158/0008-5472.CAN-11-0153 21628493 PMC3117065

[6] Nathanson DA, Gini B, Mottahedeh J, Visnyei K, Koga T, et al (2014) Targeted Therapy Resistance Mediated by Dynamic Regulation of Extrachromosomal Mutant EGFR DNA. Science 343: 72–76. 10.1126/science.1241328 24310612 PMC4049335

[7] Hoff Von DD, McGill JR, Forseth BJ, Davidson KK, Bradley TP, et al (1992) Elimination of extrachromosomally amplified MYC genes from human tumor cells reduces their tumorigenicity. Proc Natl Acad Sci U S A 89: 8165–8169. 1518843 10.1073/pnas.89.17.8165 PMC49877

[8] Kaufman RJ, Brown PC, Schimke RT (1981) Loss and stabilization of amplified dihydrofolate reductase genes in mouse sarcoma S-180 cell lines. Mol Cell Biol 1: 1084–1093. 10.1128/MCB.1.12.1084 7346713 PMC369735

[9] Mittendorf EA, Wu Y, Scaltriti M, Meric-Bernstam F, Hunt KK, et al (2009) Loss of HER2 amplification following trastuzumab-based neoadjuvant systemic therapy and survival outcomes. Clin Cancer Res 15: 7381–7388. 10.1158/1078-0432.CCR-09-1735 19920100 PMC2788123

[10] Niikura N, Liu J, Hayashi N, Mittendorf EA, Gong Y, et al (2012) Loss of Human Epidermal Growth Factor Receptor 2 (HER2) Expression in Metastatic Sites of HER2-Overexpressing Primary Breast Tumors. J Clin Oncol 30: 593–599. 10.1200/JCO.2010.33.8889 22124109 PMC3295557

[11] Wolff AC, Hammond MEH, Hicks DG, Dowsett M, McShane LM, et al (2013) Recommendations for Human Epidermal Growth Factor Receptor 2 Testing in Breast Cancer: American Society of Clinical Oncology/College of American Pathologists Clinical Practice Guideline Update. J Clin Oncol 31: 3997–4013. 10.1200/JCO.2013.50.9984 24101045

[12] Parra-Palau JL, Morancho B, Peg V, Escorihuela M, Scaltriti M, et al (2014) Effect of p95HER2/611CTF on the Response to Trastuzumab and Chemotherapy. JNCI Journal of the National Cancer Institute 106: dju291 10.1093/jnci/dju291/-/DC1 25253614 PMC4271027

[13] Marangoni E, Vincent-Salomon A, Auger N, Degeorges A, Assayag F, et al (2007) A new model of patient tumor-derived breast cancer xenografts for preclinical assays. Clin Cancer Res 13: 3989–3998. 10.1158/1078-0432.CCR-07-0078 17606733

[14] Pedersen K, Angelini PD, Laos S, Bach-Faig A, Cunningham MP, et al (2009) A naturally occurring HER2 carboxy-terminal fragment promotes mammary tumor growth and metastasis. Mol Cell Biol 29: 3319–3331. 10.1128/MCB.01803-08 19364815 PMC2698729

[15] Gianni L, Eiermann W, Semiglazov V, Manikhas A, Lluch A, et al (2010) Neoadjuvant chemotherapy with trastuzumab followed by adjuvant trastuzumab versus neoadjuvant chemotherapy alone, in patients with HER2-positive locally advanced breast cancer (the NOAH trial): a randomised controlled superiority trial with a parallel HER2-negative cohort. The Lancet 375: 377–384. 10.1016/S0140-6736(09)61964-4 20113825

[16] Xuan Q, Ji H, Tao X, Xu Y, Zhang Q (2015) Quantitative assessment of HER2 amplification in HER2-positive breast cancer: its association with clinical outcomes. Breast Cancer Res Treat 150: 581–588. 10.1007/s10549-015-3334-2 25762478

[17] Yu L, Zhao Y, Quan C, Ji W, Zhu J, et al (2013) Gemcitabine Eliminates Double Minute Chromosomes from Human Ovarian Cancer Cells. PLoS ONE 8: e71988 10.1371/journal.pone.0071988.s004 23991020 PMC3750019

[18] Sahlberg KK, Hongisto V, Edgren H, Mäkelä R, Hellström K, et al (2013) The HER2 amplicon includes several genes required for the growth and survival of HER2 positive breast cancer cells. Molecular Oncology 7: 392–401. 10.1016/j.molonc.2012.10.012 23253899 PMC5528495

