# Supplementary figures and images for: Patterns of HER2 Gene Amplification and Response to Anti-HER2 Therapies
Source: PLoS One. 2015 Jun 15;10(6):e0129876. doi: 10.1371/journal.pone.0129876 (PMC4467984; doi:10.1371/journal.pone.0129876)

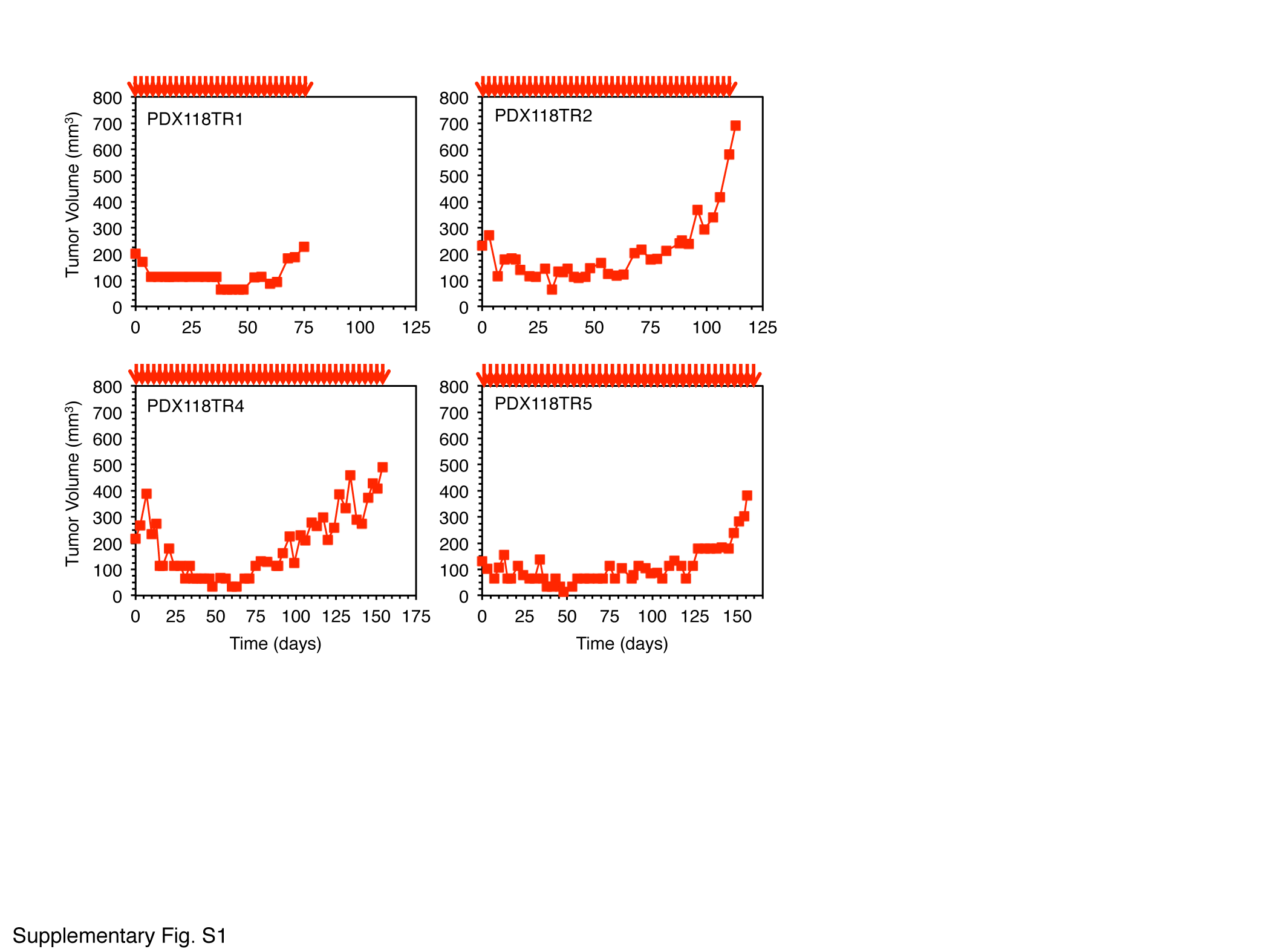

Supplement: S1 Fig — Mice carrying PDX118 were chronically treated with trastuzumab and tumor volumes were calculated as in Fig 3A. The growth of individual tumors is shown. (TIF) [file pone.0129876.s001.tif]

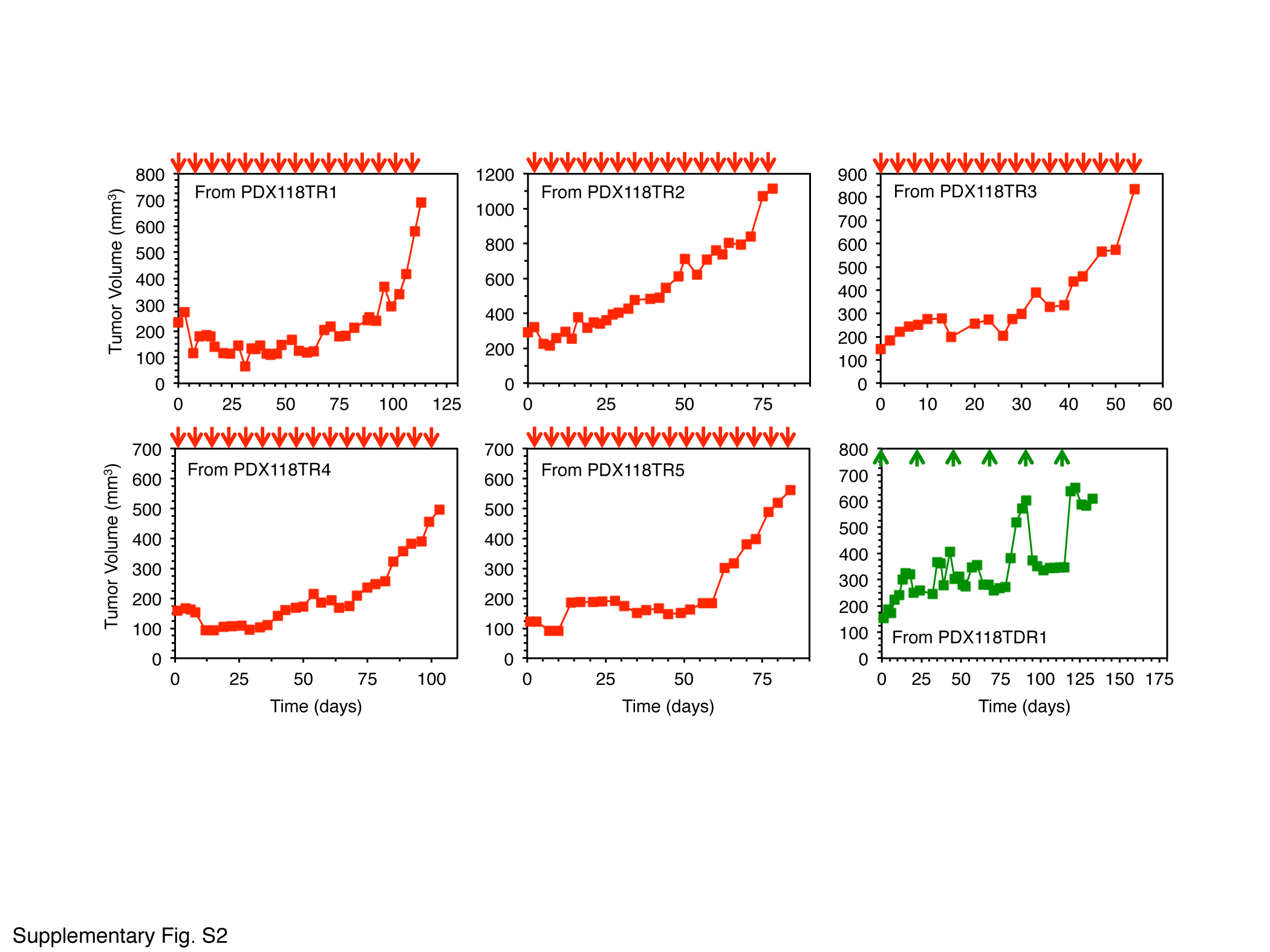

Supplement: S2 Fig — Small pieces of the tumors that progressed after trastuzumab or T-DM1 treatment (see Fig 3B and S1 Fig) were orthotopically implanted into new NOD/SCID mice (n = 3 per group). Tumor volumes were determined at the indicated time points. (TIF) [file pone.0129876.s002.tif]

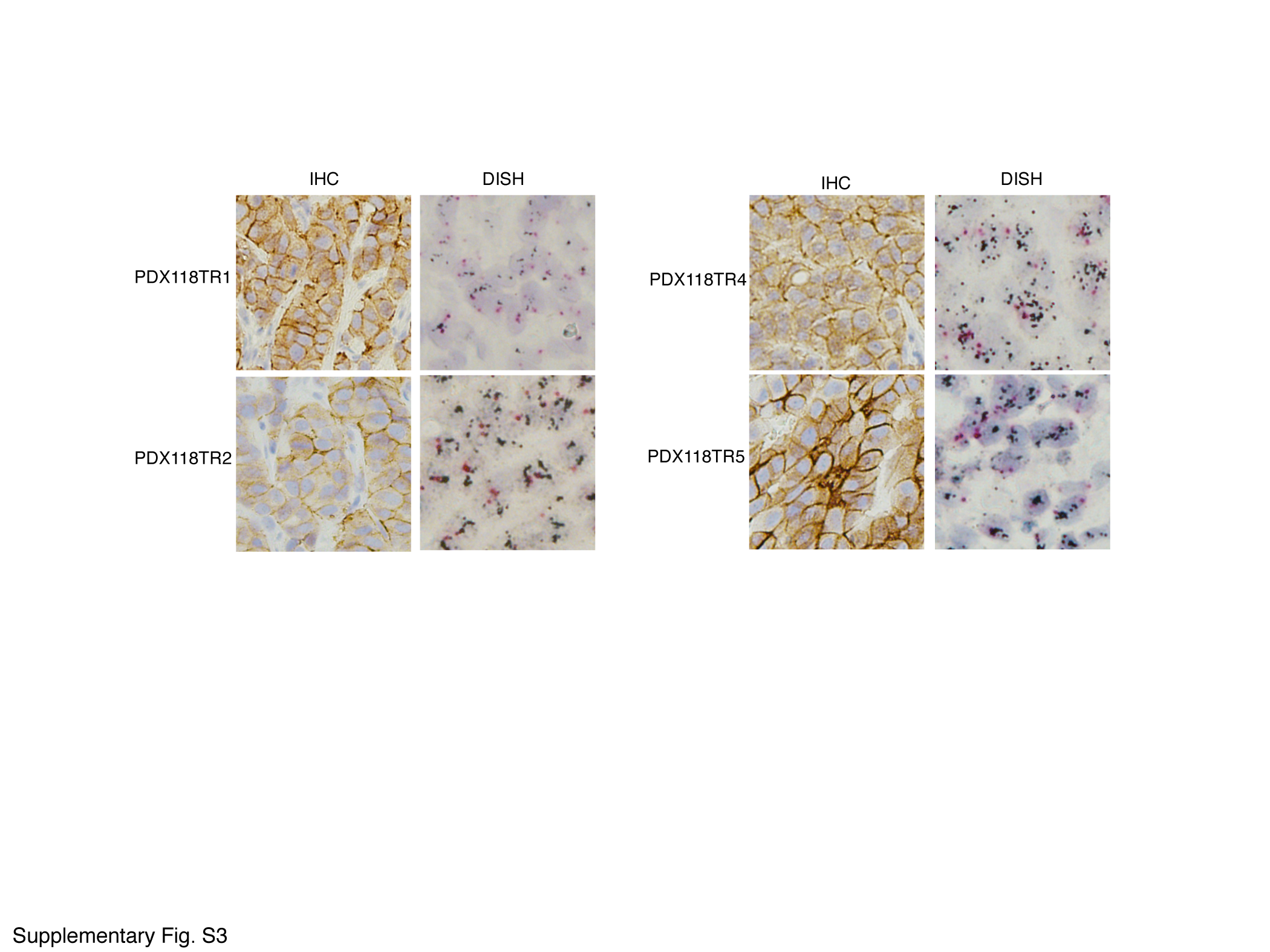

Supplement: S3 Fig — Samples from the indicated tumor grafts were analyzed by immunohistochemistry with antibodies against HER2 to determine HER2 protein levels, or by DISH to determine HER2 gene amplification. (TIF) [file pone.0129876.s003.tif]
