# Supplementary material for: Patterns of HER2 Gene Amplification and Response to Anti-HER2 Therapies
Source: PLoS One. 2015 Jun 15;10(6):e0129876. doi: 10.1371/journal.pone.0129876 (PMC4467984; doi:10.1371/journal.pone.0129876)
Supplement: S1 Table — (DOCX) [file pone.0129876.s004.docx]

S1 Table. Baseline characteristics of the cohort treated with neoadjuvant trastuzumab

| Parameter | DM | | HSR | | Mixed | |
| --- | --- | --- | --- | --- | --- | --- |
|  | No. | % | No. | % | No. | % |
| Age |  |  |  |  |  |  |
| media | 51.35 |  | 49.47 |  | 57.71 |  |
| range | 36-81 |  | 22-79 |  | 39-93 |  |
|  |  |  |  |  |  |  |
| Histological subtype |  |  |  |  |  |  |
| Ductal | 19 | 95 | 30 | 93.8 | 6 | 85.7 |
| Lobular | 0 | 0 | 0 | 0 | 0 | 0 |
| Other | 1 | 5 | 2 | 6.3 | 0 | 0 |
| Missing | 0 | 0 | 0 | 0 | 1 | 14.3 |
|  |  |  |  |  |  |  |
| Histological grade |  |  |  |  |  |  |
| G1 | 0 | 0 | 1 | 3.1 | 0 | 0 |
| G2 | 7 | 35 | 15 | 46.9 | 2 | 28.6 |
| G3 | 12 | 60 | 14 | 43.8 | 4 | 57.1 |
| Missing | 1 | 5 | 2 | 6.3 | 1 | 14.3 |
|  |  |  |  |  |  |  |
| HR status |  |  |  |  |  |  |
| ER |  |  |  |  |  |  |
| positive | 17 | 85 | 20 | 62.5 | 3 | 42.9 |
| negative | 3 | 15 | 12 | 37.5 | 3 | 42.9 |
| Missing | 0 | 0 | 0 | 0 | 1 | 14.3 |
| PR |  |  |  |  |  |  |
| positive | 12 | 60 | 17 | 53.1 | 1 | 14.3 |
| negative | 8 | 40 | 15 | 46.9 | 5 | 71.4 |
| Missing | 0 | 0 | 0 | 0 | 1 | 14.3 |
|  |  |  |  |  |  |  |
| Ki67 |  |  |  |  |  |  |
| <20% | 3 | 15 | 10 | 31.3 | 0 | 0 |
| >20% | 17 | 85 | 21 | 65.6 | 6 | 85.7 |
| Missing | 0 | 0 | 1 | 3.1 | 1 | 14.3 |
