# Supplementary material for: Patterns of HER2 Gene Amplification and Response to Anti-HER2 Therapies
Source: PLoS One. 2015 Jun 15;10(6):e0129876. doi: 10.1371/journal.pone.0129876 (PMC4467984; doi:10.1371/journal.pone.0129876)
Supplement: S2 Table — (DOCX) [file pone.0129876.s005.docx]

S2 Table. Baseline characteristics of the cohort treated with adjuvant trastuzumab

| Parameter | DM | | HSR | | Mixed | |
| --- | --- | --- | --- | --- | --- | --- |
|  | No. | % | No. | % | No. | % |
| Age |  |  |  |  |  |  |
| media | 56,4 |  | 54,2 |  | 46,7 |  |
| range | 35-81 |  | 33-86 |  | 32-73 |  |
|  |  |  |  |  |  |  |
| Histological subtype |  |  |  |  |  |  |
| Ductal | 14 | 100 | 32 | 91.4 | 7 | 100 |
| Lobular | 0 | 0 | 2 | 5.7 | 0 | 0 |
| Other | 0 | 0 | 1 | 2.9 | 0 | 0 |
| Missing | 0 | 0 | 0 | 0 | 0 | 0 |
|  |  |  |  |  |  |  |
| Histological grade |  |  |  |  |  |  |
| G1 | 1 | 7.1 | 1 | 2.9 | 0 | 0 |
| G2 | 4 | 28.6 | 12 | 34.3 | 1 | 14.3 |
| G3 | 9 | 64.3 | 20 | 57.1 | 6 | 85.7 |
| Missing | 0 | 0 | 2 | 5.7 | 0 | 0 |
|  |  |  |  |  |  |  |
| HR status |  |  |  |  |  |  |
| ER |  |  |  |  |  |  |
| positive | 9 | 64.3 | 25 | 71.4 | 5 | 71.4 |
| negative | 5 | 35.7 | 10 | 28.6 | 2 | 28.6 |
| Missing | 0 | 0 | 0 | 0 | 0 | 0 |
| PR |  |  |  |  |  |  |
| positive | 6 | 42.86 | 17 | 48.57 | 3 | 42.86 |
| negative | 8 | 57.14 | 18 | 51.43 | 4 | 57.14 |
| Missing | 0 | 0 | 0 | 0 | 0 | 0 |
|  |  |  |  |  |  |  |
| Ki67 |  |  |  |  |  |  |
| <20% | 4 | 28.6 | 11 | 31.4 | 0 | 0 |
| >20% | 10 | 71.4 | 23 | 65.7 | 7 | 100 |
| Missing | 0 | 0 | 1 | 2,9 | 0 | 0 |
