# Supplementary material for: Patterns of HER2 Gene Amplification and Response to Anti-HER2 Therapies
Source: PLoS One. 2015 Jun 15;10(6):e0129876. doi: 10.1371/journal.pone.0129876 (PMC4467984; doi:10.1371/journal.pone.0129876)
Supplement: S3 Table — (DOCX) [file pone.0129876.s006.docx]

S3 Table. Analysis of the HER2/CEP17 ratio in samples with amplification double minutes (DM) or homogeneously staining regions (HSR) pre- and post-treatment.

|  | DM | | HSR | |
| --- | --- | --- | --- | --- |
|  | pre- | post- | pre- | post- |
|  | 6.8 | 8.6 | 13.1 | 4.9* |
|  | 5.3 | 5.4 | 12.3 | 6.2 |
|  | 4.6 | 3.7 | 9.8 | 1.2 |
|  | 4.2 | 4.7 | 9.1 | 10.2 |
|  | 3.5 | 5.2 | 8.0 | 4.88 |
|  | 3.1 | 10.0 | 7.0 | 9.3 |
|  | 3.0 | 6.5 | 5.8 | 7.3 |
|  | 2.7 | 4.3 | 4.68 | 6.1 |
| Average | 4.10 | 5.82 | 8.72 | 6.25 |
| 95% CI | 0.82 | 1.53 | 2.07 | 1.95 |

- This sample was classified as DM

NOTE: Similar results were observed when we quantified only HER2 gene copies, arguing that the tendencies observed were not due to variations in the number of centromeres.
